# Heart Rate and Heart Rate Variability Correlate with Clinical Reasoning Performance and Self-Reported Measures of Cognitive Load

**DOI:** 10.1038/s41598-019-50280-3

**Published:** 2019-10-11

**Authors:** Soroosh Solhjoo, Mark C. Haigney, Elexis McBee, Jeroen J. G. van Merrienboer, Lambert Schuwirth, Anthony R. Artino, Alexis Battista, Temple A. Ratcliffe, Howard D. Lee, Steven J. Durning

**Affiliations:** 1 Division of Cardiovascular Pathology, Johns Hopkins University School of Medicine, Baltimore, USA; 2 Department of Medicine, F. Edward Hébert School of Medicine, Uniformed Services University of The Health Sciences, Bethesda, USA; 3 Department of Medicine, F. Edward Hébert School of Medicine, Uniformed Services University of The Health Sciences, Naval Medical Center, San Diego, USA; 4 School of Health Professions Education, Maastricht University, Maastricht, The Netherlands; 5 Prideaux Centre for Research in Health Professions Education, Flinders University, Bedford Park, Australia; 6 Department of Medicine, University of Texas Health Science Center, San Antonio, USA; 7 San Antonio Uniformed Services Health Education Consortium, San Antonio, USA

**Keywords:** Physiology, Human behaviour

## Abstract

Cognitive load is a key mediator of cognitive processing that may impact clinical reasoning performance. The purpose of this study was to gather biologic validity evidence for correlates of different types of self-reported cognitive load, and to explore the association of self-reported cognitive load and physiologic measures with clinical reasoning performance. We hypothesized that increased cognitive load would manifest evidence of elevated sympathetic tone and would be associated with lower clinical reasoning performance scores. Fifteen medical students wore Holter monitors and watched three videos depicting medical encounters before completing a post-encounter form and standard measures of cognitive load. Correlation analysis was used to investigate the relationship between cardiac measures (mean heart rate, heart rate variability and QT interval variability) and self-reported measures of cognitive load, and their association with clinical reasoning performance scores. Despite the low number of participants, strong positive correlations were found between measures of intrinsic cognitive load and heart rate variability. Performance was negatively correlated with mean heart rate, as well as single-item cognitive load measures. Our data signify a possible role for using physiologic monitoring for identifying individuals experiencing high cognitive load and those at risk for performing poorly during clinical reasoning tasks.

## Introduction

Diagnostic accuracy and the precise development of a management plan are imperative to improving patient safety^1–3^. Clinical reasoning can be defined as the cognitive steps (e.g. information gathering, problem representation, generating and refining diagnostic hypotheses) leading up to and arriving at a diagnosis and a management plan^4–6^. Assessing the clinical reasoning performance, however, is challenging due to the limitations of the assessment methods, many of which do not incorporate the complexity and contextual nature of clinical reasoning as a construct^7^. Given the notion that clinical reasoning is at the heart of what it means to be a clinician^8^, it is essential that we enhance our understanding of clinical reasoning and how it can be assessed.

Cognitive load theory can be a useful explanatory theoretical lens for better understanding of when clinical reasoning is successful and when it goes wrong. Cognitive load theory posits that working memory is limited in both capacity and duration (i.e., only a few elements of information can be processed at any given time, and under realistic circumstances, are held in working memory for less than twenty seconds)^9,10^. Cognitive load refers to one’s mental effort to complete a task, and it is primarily driven by element interactivity; that is, the number of cognitive elements that are simultaneously processed in working memory. In education studies, cognitive load theory posits three types of cognitive load which are affected differently by instruction and consequently have different implications for learning and performance: intrinsic load, determined by the task’s complexity and the learner’s prior knowledge; germane load, the cognitive load of construction and automation of schemata in long-term memory; and extraneous load, generated by the manner in which information is presented to learners interfering with schema acquisition and automation^11–15^.

Some researchers have criticized^16,17^ cognitive load theory as being difficult to falsify experimentally. Indeed, several studies tested hypotheses concerning the overall cognitive load and then interpreted their results in terms of intrinsic, extraneous and germane cognitive loads. This is problematic; hypotheses should be prespecified in terms of the different types of cognitive load^15,18^. There is no a priori reason that explanatory theoretical constructs cannot be applied to create a deeper understanding of complex phenomena and provide a foundation for the formulation of more concrete hypotheses^19,20^.

## Assessing the Impact of Cognitive Load on Clinical Reasoning

Several studies have provided evidence on the reliability and the validity of self-reported measures of cognitive load^15,18^. Clinical reasoning performance may be negatively associated with high cognitive load^21^. Further, high cognitive load may contribute to context specificity; e.g., seeing two patients with the same chief complaint, symptoms and findings and yet coming to different diagnoses^22^. Nonetheless, self-reported measures of perceived cognitive load may provide an incomplete picture of cognitive load^23^. Part of the problem is that individuals may be unaware of when their cognitive load exceeds capacity^21^. This is particularly salient when the excessive cognitive load happens in the “here and now” – during the busy daily clinical practice. It is reasonable to assume that when excessive cognitive load occurs, the clinician does not have cognitive resources left to reflect on the balance between cognitive load and capacity. Thus, especially in those situations, self-reports are logically of limited practical value.

By contrast, physiologic measurements are less likely to be influenced by the limitations of an individual’s ability to self-assess cognitive load. In particular, cardiovascular measures may be suitable indices of cognitive load due to their reliability and the feasibility of continuous recording^24^. One well-established cardiovascular measure is heart rate variability. Changes in heart rate variability indicate modulation of the autonomic nervous system mainly in response to changes in blood pressure and mental stress^25^. Importantly, the performance of subjects under stress may be positively or negatively impacted by the autonomic nervous system, and so, poorly controlled autonomic tone may contribute to poor performance and be a target for intervention. The connection between heart rate variability and cognitive function has been the subject of several studies^26–30^. Although heart rate variability is commonly used as an index of autonomic nervous system activity, it remains unclear whether it is sufficiently sensitive to variations in cognitive load in education scenarios. For example, in an exploratory study of computer-based training strategies, Paas *et al*.^31^ found no correlation between cognitive load and one specific aspect of heart rate variability; however, they only looked at the spectral power in the low frequency band (i.e., spectral power of the frequency band of 0.07–0.14 Hz), did not parse out different components of cognitive load, and did not include an orthogonal measure of the impact of cognitive load on the autonomic nervous system. For further review of the studies investigating the use of physiologic measures, particularly heart rate variability, to assess stress and mental workload, please see refs^32–34^.

Furthermore, research conducted in other domains suggests that biological changes may precede cognitive awareness when individuals are struggling with their thought processes (i.e., high cognitive load); for example, among professional gamblers, high sympathetic tone, as measured by skin galvanic response, was observed before these professionals could vocalize a problem with a fixed card deck^35^. We therefore specifically sought to explore if this phenomenon is present in the context of clinical reasoning as improving physician’s awareness of when help is needed could dramatically improve care and reduce error.

The purpose of this exploratory study is first to determine whether cardiovascular measures can be used as markers for cognitive load and, second, to investigate whether the more feasible option of self-report measures have biological validity evidence for clinical reasoning performance in medical students. Here, in addition to measuring the spectral power in different frequency bands, we use time-domain measures of heart rate variability; i.e., the root mean square of differences of successive heartbeat intervals (RMSSD), and the standard deviation of the normal to normal heart beat intervals (SDNN), which assesses total variability and makes no prior assumptions about the specific frequency band likely to be affected^25^. Moreover, we also measure the total variability of the QT interval (the period between the beginning of the Q wave and the end of the T wave in each cycle of the ECG signal) as an orthogonal index of the impact of cognitive load on the autonomic nervous system. This measure of QT variability is an index of the effects of changes in autonomic tone on the heart rhythm. Because they are objective and reliable^24^, physiologic markers could potentially provide an effective means to investigate the validity of self-reported measures of cognitive load.

We predicted that our findings would not only detect an association between cognitive load and clinical reasoning performance consistent with our theoretical framework, but also that there would be an association between cognitive load measures and sympathetic tone, providing additional evidence for the validity of cognitive load self-reported measures. We further predicted that these associations would be detectable during three episodes of relatively mundane clinical reasoning and not be restricted to extraordinarily challenging encounters.

## Methods

### Participants

Fifteen third- and fourth-year medical students from the Uniformed Services University of the Health Sciences were recruited to view three videos depicting physician-patient interactions and then complete a post-encounter form (PEF) for each one. Their ECG was recorded using a Holter monitor starting 24 hours before (baseline) and while they watched the videos and reported their clinical reasoning (test). Holter data for five of the participants were excluded from analysis for the following reasons: for one participant, the recording was too noisy; for two, the time stamps were not available; and for two, the data were not recorded for the full period of the experiment. The data of the remaining 10 participants were used in the analysis. There were no exclusion criteria.

### Assessment of clinical reasoning performance

As a first step, several authors crafted a written script for three video-based cases. The cases were then reviewed by an expert panel of eight internal medicine physicians and modifications were made to the script. Video cases were then filmed and re-reviewed by the same expert panel of eight internal medicine physicians for consistency.

Next, the PEF scoring rubric was constructed based on the script by having the authors generate answers for the different sections of the PEF. This was followed by review of the answers by the entire panel of experts. Following two rounds of reviews, we were able to establish complete consensus for correct, partially correct, and incorrect responses for each section. After having participants complete the PEF, additional answer options were generated that were not a part of the key (note: less than 2% of answers were not on the original key). These answers were reviewed by four of the study authors and complete consensus was reached for final responses. Reliability and validity evidence for using PEF has been collected previously^36^.

### Procedures

After informed consent, a trained researcher fitted participants with a 12-lead Holter recorder 24 hours prior to the test to establish a baseline reading. Following the 24-hour baseline period, participants were asked to sit behind a computer desk and view three outpatient clinical encounter videos that had previously undergone expert review. The first video portrayed a diagnosis of an acute retroviral syndrome, the second patient presented with colorectal cancer and an acute pulmonary embolism, and the third patient presented with new onset diabetes. The second case video, representing a life-threatening presentation, was anticipated to lead to the greatest amount of cognitive load and sympathetic tone due to the acuity of the presentation. We did not include measures of empathy, anxiety, or emotional stress as these cases were typical for the work that these physicians would be expected to encounter in practice.

During the test period, for each video, participants viewed the video and then completed the PEF followed by a single-item cognitive load rating scale. Participants then immediately re-watched the video and were asked to explain their reasoning orally using a think-aloud protocol that is similar to cued retrospective reporting^37^. Following these steps, participants completed a 10-item cognitive load measure one time at the end of the test.

### Cognitive load measures

After completion of each PEF, participants provided a self-reported single-item cognitive load measure^31^. For this, they rated their level of cognitive load exerted on the task using a Likert-type scale ranging from 1 (no cognitive load exerted) to 9 (very high cognitive load). This single-item measure is brief and has been used in several prior studies^38^.

An additional self-reported measure of cognitive load was given to each participant at the end of the three cases. It consisted of a 10-item questionnaire designed to measure the three different types of cognitive load (extraneous, germane, and intrinsic). We included these measures given the reported limitations of the single-item cognitive load measure^39^. All questionnaire items use an 11-point Likert-type scale that ranged from 0 to 10, with higher scores indicating higher cognitive load. Validity of the scores on this questionnaire as a psychometric measure has been shown in domains outside medical education^15,18^.

### Physiologic measures

ECG recordings were obtained using a high-resolution (1 kHz), digital, 12-lead, portable Holter monitoring system (Mortara Instrument Inc., Milwaukee, WI) starting 24 hours prior to the test and during the intervention. Several time and frequency domain measures were extracted from each participant’s ECG according to established guidelines^25^. Time domain measures consisted of the mean heart rate (HR, beats/min), heart rate variability calculated as the standard deviation of the time between normal beats (SDNN, msec) and root mean square of successive differences of heartbeat intervals (RMSDD, msec). The power of heart rate variability time series was measured in three frequency bands: very low frequency (VLF; 0.0037–0.04 Hz), low frequency (LF; 0.04–0.15 Hz), and high frequency (HF; 0.15–0.4 Hz). LF is associated with combined vagal and sympathetic stimulations^40^ and HF is associated with vagal stimulation and the respiratory system’s effect on the heart rate^41^; therefore, these two measures are not independent.

On average, each task took 7.01 ± 2.13 min (mean ± standard deviation), and the shortest task across all participants lasted 4.5 minutes. Therefore, to account for all the tasks in the test, the analysis was performed on 4.5-minute segments of the ECG signal, using a moving window at 0.5-minute steps. For each task, we used the average of the parameters calculated for each of the windows covered during that task. For example, VLF reported for a 7-min task is the average of VLF calculated for each of the six 4.5-min windows covered during that task. This would improve parameter estimates and lower distortion.

The QT interval was measured using a semi-automated, template matching algorithm that has been previously described^42^. Briefly, the algorithm generates several signal-averaged templates from a chosen ECG lead. For each template, the investigator identifies a representative complex, including the entire QT and U wave in order to include all components related to depolarization and repolarization of the ventricles. The inclusion of the U wave has been previously shown to improve the predictive value of the metric for life-threatening arrhythmias^43^. Each individual QT interval value is then calculated as how much each beat needs to be stretched or compressed to fit the corresponding template QT. A normalized QT variability index (QTVI) was also derived according to the following equation:

$${\rm{Q}}{\rm{T}}{\rm{V}}{\rm{I}}={\log }_{10}[({\rm{Q}}{\rm{T}}{\rm{v}}/{{\rm{Q}}{\rm{T}}}^{2})/({\rm{H}}{\rm{R}}{\rm{v}}/{{\rm{H}}{\rm{R}}}^{2})],$$

where HR = mean heart rate, HRv = heart rate variance, QT = mean QT interval, and QTv = QT interval variance. QTVI formula is designed to produce an independent measure by including QT and HR (which are not independent) in the numerator and the denominator.

To limit the effect of posture or physical activity on the physiologic measures, the participants were asked to keep sitting as they watched the videos, filled out the questionnaires, or explained their thinking process.

### Clinical reasoning performance measures

Participants’ performance for each scenario was measured using a PEF, on which they indicated a leading diagnosis, differential diagnosis, supporting data and a therapeutic management plan. Scoring of the PEF entailed having a group of experts construct and revise answer key responses through a series of discussions. Complete consensus was achieved for this scoring rubric. Reliability and validity of this PEF for the assessment of clinical reasoning has been previously established^36,44^. Each PEF consisted of the following prompts:

#### Patient history

What else do you want to ask this patient? (List one to five questions).

#### Physical exam

What else would you want to look for on this patient’s physical exam? (List one to five items).

#### Differential diagnosis

What is your differential diagnosis? (please list in order of likelihood and list at least 3 responses).

#### Supporting evidence

What data supports this diagnosis? (List one to five pieces of evidence).

#### Treatment/management plan

What is your treatment/management plan for this patient (diagnostic and/or therapeutic).

An expert panel generated scores for every entry on the PEF with complete consensus. This was achieved after two rounds of review and edits to potential PEF responses. Scores for each response ranged from 0 (incorrect), to 1 (partially correct), and 2 (correct). Scores for all responses were tallied to generate a total score for clinical reasoning performance (maximum score of 30).

### Data analysis

Correlation analysis was performed to assess the association between the self-reported cognitive load measures and physiologic measures. For this purpose, partial correlation was measured to control for gender differences in physiologic measures of heart rate variability^33^. Correlation analysis was also used to explore the relationship between clinical reasoning performance scores and cognitive load using both physiologic measures and self-reported measures of cognitive load. We extracted the time and frequency domain parameters for the time period that each participant spent watching and completing the PEF and think-aloud protocols for each video. Participants’ average physiologic measures during each task were used to calculate the correlation coefficients. Signal processing, feature extraction and data analysis were performed using in-house software developed in MATLAB^45^. Data are presented as mean ± standard error of the mean unless noted otherwise. For correlation analysis, we set type I error rate of α = 0.05. When considering each task separately, we set the minimum correlation coefficient of |ρ| ≥ 0.67. With 15 subjects, our analysis would have 80% power (i.e., type II error rate of β = 1 − power = 0.2). Because we lost data from 5 out of 15 subjects, our analysis power dropped to 60% (i.e., β = 0.4)^46^. Due to this increase in type II error, there might be associations between the cardiovascular parameters of each specific task and performance/cognitive load measures that we failed to detect; however, the type I error rate was kept low (α = 0.05).

### Ethical approval

The data were stored and analyzed anonymously, and this study was deemed exempt by IRB at Uniformed Services University of the Health Sciences. Informed consent was obtained from all participants prior to the study. All research was performed in accordance with relevant guidelines and regulations.

### Disclaimer

The views expressed in this paper reflect the opinions of the authors only and not the official policy of the United States Army, Uniformed Services University, or the Department of Defense.

## Results

### Study cohort

The final sample of ten participants contained 2 females, and the mean age was 25. None were on any prescribed medications. Average electrocardiographic variables recorded at baseline (24-hour period preceding the test) and during the test are reported in Table 1. For each case, participants took 5.6 ± 0.2 min to watch the video, 8.97 ± 0.32 min to fill out the PEF, and 6.5 ± 0.33 min for think-aloud.

**Table 1:** Baseline mean values of the physiologic parameters measured 24 hours prior to the test.

	HR (beat/min)	SDNN (msec)	QTVI
24 hours prior to the test	71.04 ± 2.73	79.11 ± 5.94	−1.42 ± 0.06
During the test	68.88 ± 2.70	70.32 ± 3.80	−1.46 ± 0.10

The parameters are reported as mean ± standard error of the mean.

### Cognitive load and clinical reasoning performance

Participants’ performance scores on the PEF ranged from 11 to 25 (17 ± 1.73) for the first video, 16 to 27 (22.1 ± 1.29) for the second video, and 10 to 25 (16.7 ± 1.57) for the third video. The average single-item measures of cognitive load were 5.9 ± 0.53 after the first (CL1), 6.5 ± 0.4 after the second (CL2), and 7.4 ± 0.31 after the third video (CL3), showing a steady increase (CL3 > CL1, *p < *0.05). On the 10-item inventory, intrinsic, germane and extraneous types of cognitive load were measured: scores for intrinsic and germane cognitive loads ranged from 3 to 8 (4.97 ± 0.55 and 5.03 ± 0.52, respectively), and scores for extraneous cognitive load ranged from 0 to 10 (2.07 ± 0.98).

Across all three case videos (n = 30), performance scores negatively correlated with single-item measures of cognitive load (r = −0.47, *p* < 0.01). However, we did not find any statistically significant correlation between the 10-item measures of the three different types of cognitive load and performance scores.

### Cognitive load and cardiovascular measures

**Figure 1:**
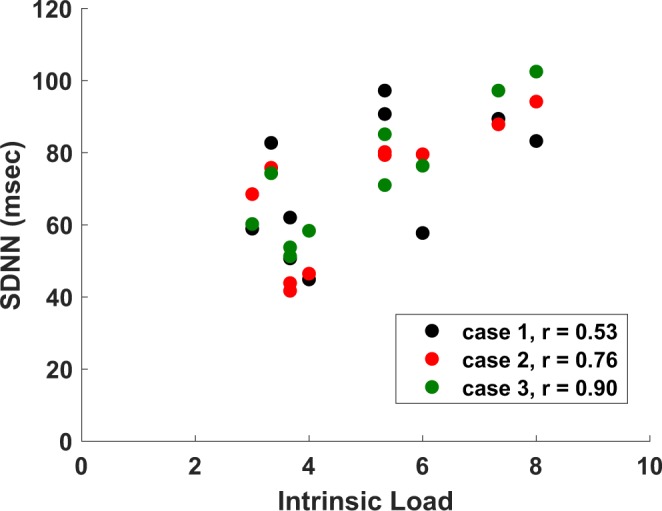
Scatterplot of SDNN versus intrinsic cognitive load during the think-aloud sessions of the three clinical cases. The correlation between intrinsic cognitive load and SDNN increased during the experiment.

Here, we assessed the correlation between measures of cognitive load (intrinsic, germane, and extraneous) and cardiovascular measures. During the test, intrinsic cognitive load was positively correlated with heart rate variability features in both time and frequency domains, including SDNN, RMSSD, LF and VLF power (Table 2). Of note, the correlation between self-reported intrinsic cognitive load and SDNN measured during think-aloud sessions increased across the three video tasks (Fig. 1). A steady increase was also seen in the correlation between self-reported intrinsic cognitive load and LF power measured during think-aloud (Table 2). QTVI was strongly associated with single-item measures of cognitive load during the second case video (Table 2).

**Table 2:** Correlations between measures of self-reported cognitive load and physiologic measures.

Cognitive Load Measure	Physiologic Measure	Correlation Coefficient	*p*-value
Intrinsic	LF	0.91	0.001
Intrinsic	SDNN	0.71	0.031
Intrinsic	RMSSD	0.69	0.040
Germane	VLF	0.68	0.045
CL1 + CL2 + CL3	QTVI	0.72	0.030
Intrinsic	t1 LF	0.70	0.035
Intrinsic	v2 LF	0.77	0.016
Intrinsic	v2 QT	0.75	0.033
Intrinsic	p2 LF	0.72	0.028
Intrinsic	p2 RMSSD	0.71	0.032
Intrinsic	t2 SDNN	0.76	0.019
Intrinsic	t2 VLF	0.73	0.027
Intrinsic	t2 LF	0.73	0.026
Intrinsic	v3 LF	0.73	0.026
Intrinsic	p3 LF	0.74	0.022
Intrinsic	p3 RMSSD	0.74	0.023
Intrinsic	t3 SDNN	0.90	0.001
Intrinsic	t3 VLF	0.76	0.018
Intrinsic	t3 LF	0.90	0.001
Intrinsic	t3 HF	0.72	0.030
Intrinsic	t3 RMSSD	0.86	0.003
Germane	p1 SDNN	0.84	0.005
Germane	p1 VLF	0.82	0.007
Germane	p1 LF	0.72	0.028
CL2	p2 QTVI	0.81	0.008
CL2	t2 QTVI	0.77	0.016
CL2	p3 QTVI	0.89	0.001
CL2	t3 QTVI	0.81	0.008
CL3	p3 RMSSD	0.69	0.040

v*n*, p*n* and t*n* indicate the physiologic measures averaged during watching, PEF completion and the think-aloud sessions for clinical case *n* (1–3), respectively. When task number is not indicated, the full test period (63.1 ± 1.87 min) was used for the measurement.

### Clinical reasoning performance and cardiovascular measures

Table 3 lists the statistically significant correlations of clinical reasoning performance scores with cardiovascular measures. Performance scores for clinical case 2 were positively correlated with heart rate variability as measured by SDNN and VLF while the participants watched the video. Those with better performance for case 2, manifested lower heart rates and higher QT interval during the following task. These correlations were not present for the other two cases. For case 3, the performance score was negatively correlated with QTVI while the participants watched the video.

**Table 3:** Correlations between objective performance measures and cardiovascular variables.

PEF-based Performance Measure	Physiologic Measure	Correlation Coefficient	*p*-value
Case 2 Performance Score	v2 SDNN	0.69	0.042
	v2 VLF	0.68	0.042
	v2 QT	0.71	<0.05
	v3 QT	0.68	0.045
	p3 HR	−0.70	0.037
	p3 QT	0.69	0.042
	t3 QT	0.73	0.026
Case 3 Performance Score	v3 QTVI	−0.73	0.024

v*n*, p*n* and t*n* indicate the physiologic measures averaged during watching the video, PEF completion and the think-aloud sessions for case *n* (1–3), respectively.

## Discussion

The major findings of this study were threefold: (a) we found strong correlations between cardiovascular measures and self-reported measures of cognitive load during clinical reasoning; (b) performance scores negatively correlated with single-item measures of cognitive load; and (c) we found strong negative correlations between objective measures of performance and mean heart rate for one task. QT duration was also correlated with performance, but this effect likely reflects the same phenomenon as heart rate, i.e. an increase in sympathetic tone. The correlations between performance and the physiologic measures reported in this study were not present with the physiologic measurements 24 hours prior and were only present on the test day. These findings were consistent with our hypothesis that high cognitive load would correlate with physiologic measures of sympathetic tone. The correlations were strongest for case 2, which represented the most urgent presentation (a patient with colorectal cancer and a pulmonary embolism), as the acuity of this life-threatening presentation would be expected to invoke greater sympathetic tone. This finding could have significant implications for the assessment of individuals performing complex tasks that are associated with significant failure risks.

Our analysis showed a positive correlation between intrinsic cognitive load and heart rate variability frequency and time domain measurements (Table 2). An increase in heart rate variability is generally regarded as an indication of a robust autonomic nervous and cardiovascular system^25^, and it is somewhat surprising that it was associated with increased cognitive load. Heart rate variability can increase due to an increase in parasympathetic or sympathetic tone (or both)^40^. Our findings suggest that an increase in perceived cognitive load appears to result in an increase in both sympathetic and parasympathetic components of the autonomic nervous system. While mental stress is typically associated with decreased parasympathetic tone, an increase in blood pressure may have had the opposite effect in our cohort. Mental stress has been shown to increase blood pressure^47^, and an increase in blood pressure in young healthy individuals could be expected to increase parasympathetic tone via the baroreceptor reflex mechanism.

QTVI, a validated measure predicting adverse cardiovascular events, was correlated with self-reported single-item measures of cognitive load overall, and particularly at the end of the second case, as well as the performance scores for the third case. In healthy individuals, heart rate and QT interval are inversely correlated; as heart rate increases, the QT interval shortens. Activation of the sympathetic nervous system and parasympathetic withdrawal significantly increases heart rate and shortens the QT interval through direct and indirect effects on the myocardium. QTVI is a log ratio of normalized QT variance over normalized heart rate variance, and therefore an increase in QTVI in the setting of increased heart rate variability is somewhat unusual, indicating that repolarization variability increased to a greater extent than heart rate variability. Identifying trainees who are experiencing increased cognitive load could have important implications for physician health and for program level wellness initiatives.

Clinician’s performance is a critical concern to patients and health systems, and identifying clinicians that are in danger of clinical reasoning performance failure prior to making an error is an important goal. In this study, we found a strong inverse correlation between heart rate and an objective performance score during a clinical reasoning exam, indicative of activation of the sympathetic nervous system in those at risk of doing poorly. In addition, self-reports of cognitive load are not feasible to be used during normal clinical practice, whereas these are the contexts in which this balance between cognitive load and capacity may be most detrimental. If a clinician is overwhelmed at times by the situational demands, they generally do not have the time to sit and think, or reflect, or take a ‘timeout’ in every situation. The understanding of the relationship between cognitive load, risk of underperformance and physiological parameters may be useful to design monitoring warning instruments for practicing clinicians in complex settings to enhance self-monitoring – a critical component of self-regulation.

The current study is unique in that it bridges multiple fields: cognitive psychology, physiology, and medicine. It is a first attempt to measure clinical reasoning performance using the proxy of cognitive load with physiologic parameters that are not subject to error in self-reports. As stated in the introduction, all assessments bear in them the problem of having to infer mental processes from observing external behavior and this inference is always influenced by the validity evidence in the context of current validity theory. Physiological parameters could potentially serve as a more direct measurement of cognitive load. Therefore, the findings from this study may have important practical significance and implications in medical education, especially with respect to the development of tools to optimize the influence of cognitive load and improve clinical reasoning performance. The increasing use of personalized monitors for heart rate and even electrocardiogram makes it likely that these findings could be potentially employed to monitor trainees to optimize their clinical reasoning ability, as well as their personal health and to preempt clinical failure.

This study also had several limitations. First, the sample of participants in this study was quite small. Out of original 15, five participants’ data had to be excluded for technical reasons. However, the identified effect sizes were large, and the results were statistically significant. Second, the study was conducted in a low-stakes experimental environment, which might have attenuated the effects of cognitive load on performance. Third, the absence of blood pressure as a gauge of physiologic response to stress limits any inferences we might have been able to make regarding its potential moderating role on the impact of cognitive load on performance. Fourth, we did not explore the learning process in this investigation, and there may be differential effects on learning and performance in trainees in terms of cognitive load.

For the purposes of our analysis, we have applied the prevalent assumption that the autonomic nervous system – and the indices of heart rate variability and QTVI – represent purely reactive phenomena triggered by the perception of external stimuli. The “Polyvagal Theory”, however, suggests that there are phylogenetic differences in the organization of the parasympathetic system that support a bidirectional interaction for the autonomic system and higher behaviors^48^. In mammals, the parasympathetic system incorporates central nuclei that allow the system to not only suppress sympathetically-driven vegetative functions (i.e., blood pressure and heart rate), but to also modulate internal perceptions, facial behaviors, and ultimately social interactions^49^. Testing this hypothesis is beyond the scope of this study, but future investigations could explore the impact of parasympathetic intervention (i.e., exercise training) on perceived cognitive load and performance.

Our current findings have the potential to inform assessment of clinical reasoning performance in authentic (e.g. patient care) settings. Such work could also advance our understanding of context specificity, which leads to unwanted variation in physician performance. For example, consistent with the literature on cognitive load, instructional materials could then be developed to assist the clinician/student with reducing cognitive load and improving future performance. The inclusion of physiologic monitoring in a training regime could provide “real time” feedback to the learner regarding the effectiveness of that regime.

One implication for practice is to determine if expected increases in sympathetic tone would be seen before an individual is able to vocalize that they are dealing with a challenging situation (e.g., that they are “out of their depth”). We envision future means of looking at heart rate variability by emerging hand-held or wearable technologies to help the physician know when they may need help with clinical care, as well as using heart rate variability monitors to generate validity evidence for more common assessment measures of clinical reasoning in practice.

## Data Availability

Anonymized data are available from the corresponding author upon reasonable request.
